# Agile implementation of alcohol screening in primary care

**DOI:** 10.1186/s12875-024-02500-7

**Published:** 2024-07-11

**Authors:** Diana Summanwar, Chelan Ropert, James Barton, Rachael Hiday, Dawn Bishop, Malaz Boustani, Deanna Willis

**Affiliations:** 1 Department of Family Medicine, Indiana University School of Medicine, Indianapolis, IN 46202 USA; 2 Altarum, Ann Arbor, MI 48105 USA; 3 Department of Medicine, Indiana University School of Medicine, Indianapolis, IN 46202 USA; 4 Indiana University Center for Aging Research, Indianapolis, IN 46202 USA; 5 Sandra Eskenazi Center for Brain Care Innovation, Eskenazi Health, Indianapolis, IN 46202 USA

**Keywords:** Alcohol screening, Primary care, Agile, Implementation, Healthcare delivery, Substance use disorders, Quality improvement

## Abstract

**Background:**

Despite the United States Preventive Services Task Force recommendation to screen adults for unhealthy alcohol use, the implementation of alcohol screening in primary care remains suboptimal.

**Methods:**

A pre and post-implementation study design that used Agile implementation process to increase screening for unhealthy alcohol use in adult patients from October 2021 to June 2022 at a large primary care clinic serving minority and underprivileged adults in Indianapolis.

**Results:**

In comparison to a baseline screening rate of 0%, the agile implementation process increased and sustained screening rates above 80% for alcohol use using the Alcohol Use Disorders Identification Test – Consumption tool (AUDIT-C).

**Conclusions:**

Using the agile implementation process, we were able to successfully implement evidence-based recommendations to screen for unhealthy alcohol use in primary care.

**Supplementary Information:**

The online version contains supplementary material available at 10.1186/s12875-024-02500-7.

## Background

Alcohol use disorder is the most prevalent substance use disorder worldwide [1]. In the United States (U.S.), 29.5 million individuals aged 12 and above were reported to have alcohol use disorder (AUD) in 2021 [2, 3]. Between 2015 and 2019, excessive alcohol use accounted for over 140,000 deaths and 3.6 million years of potential life lost annually, becoming the fourth leading cause of preventable death in the United States [2, 4]. However, only 4% or approximately 1.4 million, received treatment for their condition [5]. Currently, the U.S. Preventive Services Task Force (USPSTF) recommends that individuals 18 years and older undergo screening for unhealthy alcohol use in primary care settings [6]. The USPSTF concluded that appropriate screening tools can effectively assess alcohol use and that brief counseling interventions in adults who screen positive are associated with a reduction in weekly alcohol consumption [6, 7]. Despite this recommendation, screening for unhealthy alcohol use using a validated questionnaire only occurs during 2.6% of U.S. adult primary care visits [8].

This gap between research evidence and practice is a problem widely recognized [9]. A recent study estimated that the average time to implementation of cancer-related evidence-based practices (EBP) is 15 years. A marginal improvement from a previous estimate of 17 years [10]. This “research to real world” gap arises because healthcare professionals may not feel that EBPs apply to their more complex, real-world patients or that implementing Evidence-based healthcare solutions is not feasible or too time-consuming due to real-world constraints and lack of demand for implementing EBP in their local health-care system [11, 12].

Over the past two decades, implementation science has focused on closing the research-to-practice gap by developing strategies, processes, and tools to overcome barriers, improving implementation, and speeding the timeline from evidence to practice. The agile implementation (AI) process, developed at the Indiana University Center for Health Innovation and Implementation Science (CHIIS), addresses this gap by providing a systematic process to identify and overcome challenges that may be unique to the local population and healthcare system. AI process leverages insights from behavioral economics, complex adaptive systems theory, and network sciences to understand, predict, and steer the behavior of an individual or a social organization such as a healthcare delivery system [13, 14].

Thus, the goal of this study was to assess the efficacy of an AI process in augmenting the screening rates for unhealthy alcohol use within a primary care setting.

## Methods

Setting: This project was a quality improvement (QI) study conducted at an Indiana University Health primary care clinic. The study period extended from October 2021 to July 2022. The clinic hosts 67 clinicians, which includes 10 faculty physicians, 2 advanced practice providers, 39 family medicine resident physicians, and 15 transitional year residents. During the study timeframe, the average number of adult visits per month was 2,200. The patient population breakdown is 50% Medicaid, 13% Medicare, 20% private insurance, and 16% self-pay. Racial minorities comprise 59% of the patient population.

**Fig. 1:**
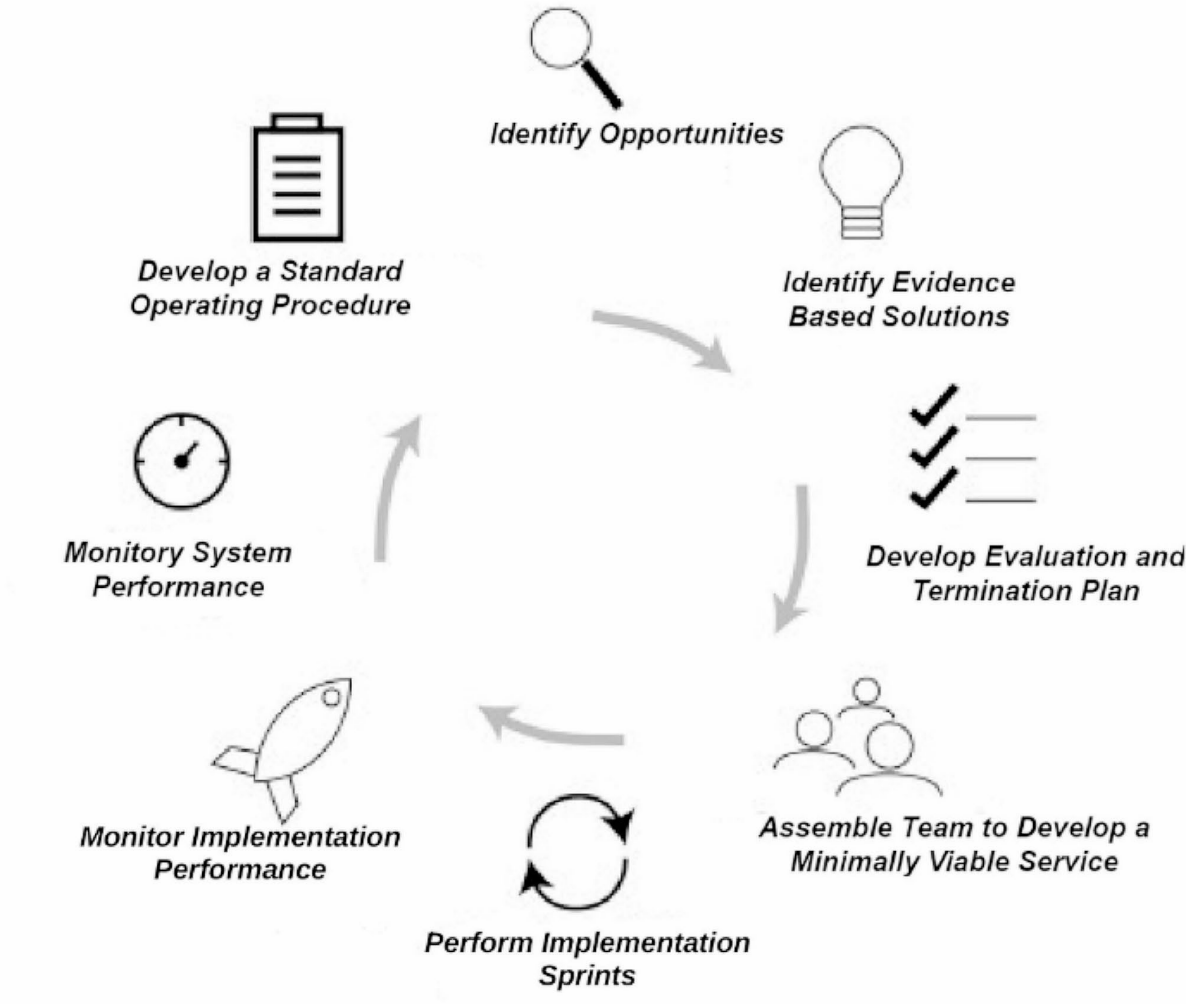
Agile implementation process

The Implementation Process: The AI process was selected as the model for improvement. AI describes a reproducible and scalable process to rapidly localize, implement, and sustain evidence-based healthcare services [15, 16]. The AI process consists of eight key steps (Fig. 1).

The first step involves identifying potential opportunities for improvement while also confirming the availability of time, personnel, and resources to address the problem. Our team accomplished this by joining the Michigan Sustained Patient Centered Alcohol Related Care (MI-SPARC) collaborative in the fall of 2021. MI-SPARC study tested whether implementation approaches shown effective in the SPARC trial—practice facilitation and EHR support—were effective in other primary care settings when accompanied by 2 h of primary care provider training [17]. Through this program, providers received instruction on recognizing and treating unhealthy alcohol use. They also received technical assistance on implementing screening, brief preventative counseling, and referral to treatment (SBIRT) in their clinic workflow.

Additionally, we established a team of volunteers interested in leading the clinic-wide implementation effort, including physicians, medical assistants, front desk team members, and social workers.

In the second step, the team identifies an evidence-based solution by referencing published studies, guidelines, or recommendations. For our initiative, evidence-based screening tools were provided by MI-SPARC. This included the Alcohol Use Disorders Identification Test (AUDIT) and its abbreviated version, the AUDIT-C. The AUDIT, developed by the World Health Organization, comprises ten questions and enquires about alcohol intake, potential dependence on alcohol, and experience of alcohol-related harm [18]. The AUDIT-C is a three-item questionnaire that comprises the first three questions of the AUDIT consumption measures. It is a practical, valid primary care screening test for heavy drinking and/or active alcohol abuse or dependence. [19].

The third step involved developing an evaluation and termination plan, which includes defining milestones, outcome measures, and the establishment of criteria for discontinuing the intervention if it proves unsuccessful. Our team defined the evaluation criteria based on the screening rates for unhealthy alcohol use using the AUDIT tool as our primary measure. Our termination criteria were set as a screening rate below 50% at nine months from the start date, with the determination to be made by our site champion.

The fourth step requires an interdisciplinary team to map the current process and identify the essential specifications of the evidence-based solution, referred to as the ‘minimally viable product or service, tailored to our unique setting. For this step, the team engaged in the customization or ‘localization’ of the AUDIT screening tool. This involved the design of a user-friendly tool that featured the AUDIT-C questions on one side, while the reverse side contained the additional seven questions of the full AUDIT. This was accompanied by a visual representation illustrating a standard drink size (Appendix 1).

Additionally, MI-SPARC conducted online educational sessions on alcohol screening, SBIRT, and pharmacological treatment. These sessions were made available in both live and recorded formats for clinicians and staff.

Step five entails the execution of time-bound implementation cycles, referred to as ’sprints’, designed to test the minimal specifications identified earlier. These sprints serve as a dynamic phase where the performance of the implementation is closely monitored (step six) using predetermined outcome measures and milestones. Simultaneously, the team evaluates the impact of the intervention on the overall organization, assessing unintended adverse and positive consequences (step seven).

Once the solution successfully meets the desired goals, the eighth step involves the development of a standardized operating procedure. This procedure serves as a blueprint for others to implement the solution across different sites. In essence, these steps collectively constitute the AI process [13].

### Study measurement

Key measures: The primary metric selected for this initiative encompassed the proportion of adult patients who underwent alcohol screening by completing the AUDIT tool during any category of visit. Additionally, we monitored the distribution of AUDIT forms as a process measure.

Sampling and Chart Review: A random sampling approach was adopted, with 40 patient charts selected for review each month. This process resulted in a cumulative total of 195 charts reviewed. The documentation within these charts was examined for evidence of AUDIT screening, which included various documentation types such as ad-hoc entries in medical records, clinician notes, or scanned copies of the AUDIT questionnaire. This approach ensured the inclusion of all screening instances, regardless of the documentation style.

### Analysis

Design and Methodology: We adopted a longitudinal time series analysis framework, with baseline data collected from the two months preceding the intervention. We monitored outcomes continuously during the active phase of the intervention and following its conclusion.

Outcome Measures: The primary outcome measure was the percentage of patients who completed the AUDIT screening. We assessed the persistence of these practices post-implementation by examining data subsequent to the clinic’s relocation to a new facility in September 2022. This analysis aimed to evaluate the long-term stability and effectiveness of the implemented procedural changes.

## Results

Our Implementation sprints began in February 2022, with the clinic-wide team convening every two weeks to review screening rates. In our workflow’s initial configuration, we tasked medical assistants (MAs) with the distribution of screening tools during patient escort to the clinic rooms. Visual cues, including signage at exam room entrances and conveniently placed AUDIT forms by computers, were introduced to nudge MAs towards screening execution. However, a post-implementation review of three cycles indicated these interventions did not improve screening rates. Consequently, we modified our approach. In the following sprint, we tested having the front desk team members provide the form to patients during the check-in process. In addition, we included a communication within the form, emphasizing that alcohol screening was an integral component of comprehensive care for all adults visiting the clinic. We changed the frequency of our team meetings to weekly, lasting only 15 min, with at least one representative from each clinic team present. During these meetings, we reviewed screening rates using a visual run chart and addressed the following questions:

1. What worked well?
2. What didn’t work well?
3. What adjustments do we plan to make based on what we learned?

Each team member discussed the feedback on screening rates, shared a patient story from the previous week, and outlined the planned changes for the upcoming week. The communication with the rest of the members occurred during huddles, staff meetings, pod meetings, and through group text messages.

**Fig. 2:**
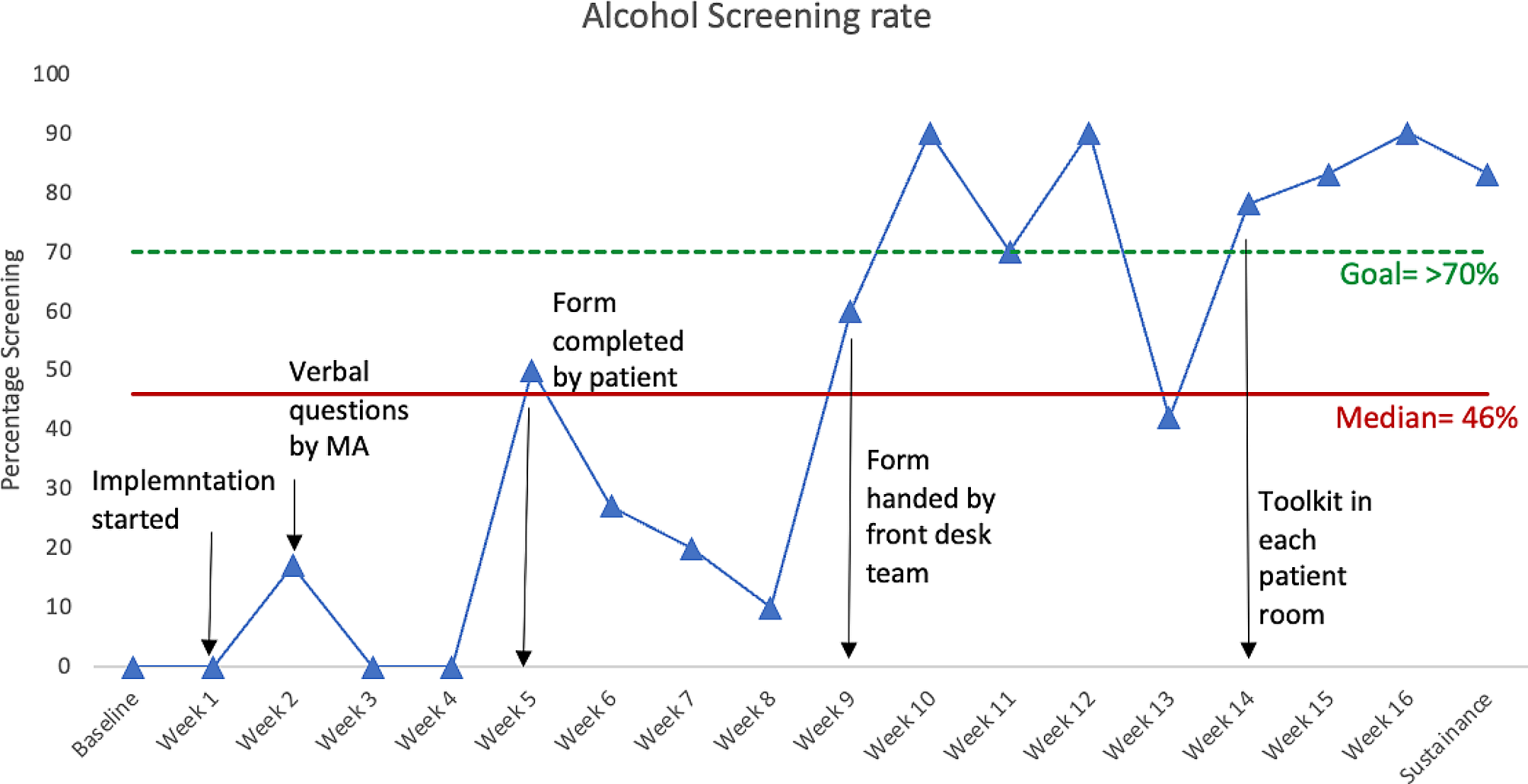
Alcohol screening rate with the AUDIT tool

The screening rate for identifying unhealthy alcohol use, assessed through a validated tool, began at a baseline of 0%. However, during the initial implementation phase, when medical assistants verbally asked the screening questions to the patients after the rooming process, the screening rate consistently fell below the median line of 46%. During debriefing sessions, clinical staff expressed concerns about potential patient discomfort when asked about their drinking habits. This led to the development of an improvement cycle (sprint) aimed at exploring the feasibility of having patients independently complete the AUDIT tool, eliminating the need for verbal inquiries from the clinical staff. This change yielded only a single data point above the median. Subsequent cycles prompted a modification in the process, where the front desk team provided the screening tool to patients during the check-in process. This resulted in a series of data points above the median, reaching screening rates of 90%. Fewer runs than anticipated, as indicated by tabled critical values, indicated the presence of non-random variation within the process [20]. During the sustainability period, the screening rate remained at 83%, significantly higher than during the baseline period (Fig. 2).

Additionally, other identified barriers were the recognition of knowledge gaps regarding specific concepts related to alcohol screening and brief interventions, such as drinking limits, the content of brief interventions, local resources, and pharmacological treatment. To address these barriers and enhance the screening process, using tools and resources provided by MI-SPARC, the team developed a provider tool kit. This tool kit encompassed a decision tree, brief interventions, patient education brochure, a list of community resources and referrals, as well as a table outlining pharmacological therapeutic options. Through multiple iterations based on the feedback of the clinicians, the provider tool evolved from a paper file kept in each patient room to a website accessible via a QR code embedded on the screening form.

## Discussion

Using the AI process, our team effectively identified and implemented evidence-based solutions for screening adults for unhealthy alcohol use. The implementation led to a substantial 90% increase in screening rates using a validated tool. We attribute the success and sustainability of our screening efforts to the active involvement of physicians, medical assistants, and front desk staff throughout the process, alongside a focus on shared learning and collaboration, the establishment of psychological safety, regular accountability meetings with transparent data updates, and leadership support. The development of termination plans for each sprint not only facilitated evaluation, feedback, and continuous learning but also promoted an iterative development approach. Furthermore, it played a key role in keeping the team detached from any particular solution, enabling them to direct their focus toward achieving the desired outcome.

The development of a standardized operating procedure that employs the AUDIT tool’s predefined templates integrated in our electronic medical system will serve as a blueprint for multiple primary care clinics. Future steps should be taken to standardize data collection and analysis processes across these clinics, allowing for a comprehensive evaluation of the implemented practices’ impact on patient outcomes.

Current literature underscores the complexities associated with alcohol screening in primary care settings, particularly in the context of real-world clinical adherence [21–23]. It is clear that strategic and effective implementation plays a critical role in achieving best practice standards and optimizing patient outcomes. The AI process provides a comprehensive framework for successfully and sustainably implementing unhealthy alcohol use.

It is important to acknowledge several limitations when interpreting our study’s results. Firstly, the data collection process was limited to a subset of eligible patients, involving a review of a limited number of charts. Additionally, our study did not capture information regarding interventions and referrals for patients with positive screenings. Consequently, it remains unclear whether our approach effectively facilitated interventions.

## Conclusions

By leveraging the AI process, we successfully implemented evidence-based recommendations for screening for unhealthy alcohol use at our academic primary care clinic. The integration of increased communication and interprofessional collaboration proved integral to enhancing care and sustaining improvements over time.

### Electronic supplementary material

Below is the link to the electronic supplementary material.

Appendix 1. Adapted AUDIT tool

## Data Availability

The data that support the findings of this study are available on request from the corresponding author, DS.
