# Supplementary figures and images for: Agile implementation of alcohol screening in primary care
Source: BMC Prim Care. 2024 Jul 11;25:251. doi: 10.1186/s12875-024-02500-7 (PMC11238453; doi:10.1186/s12875-024-02500-7)

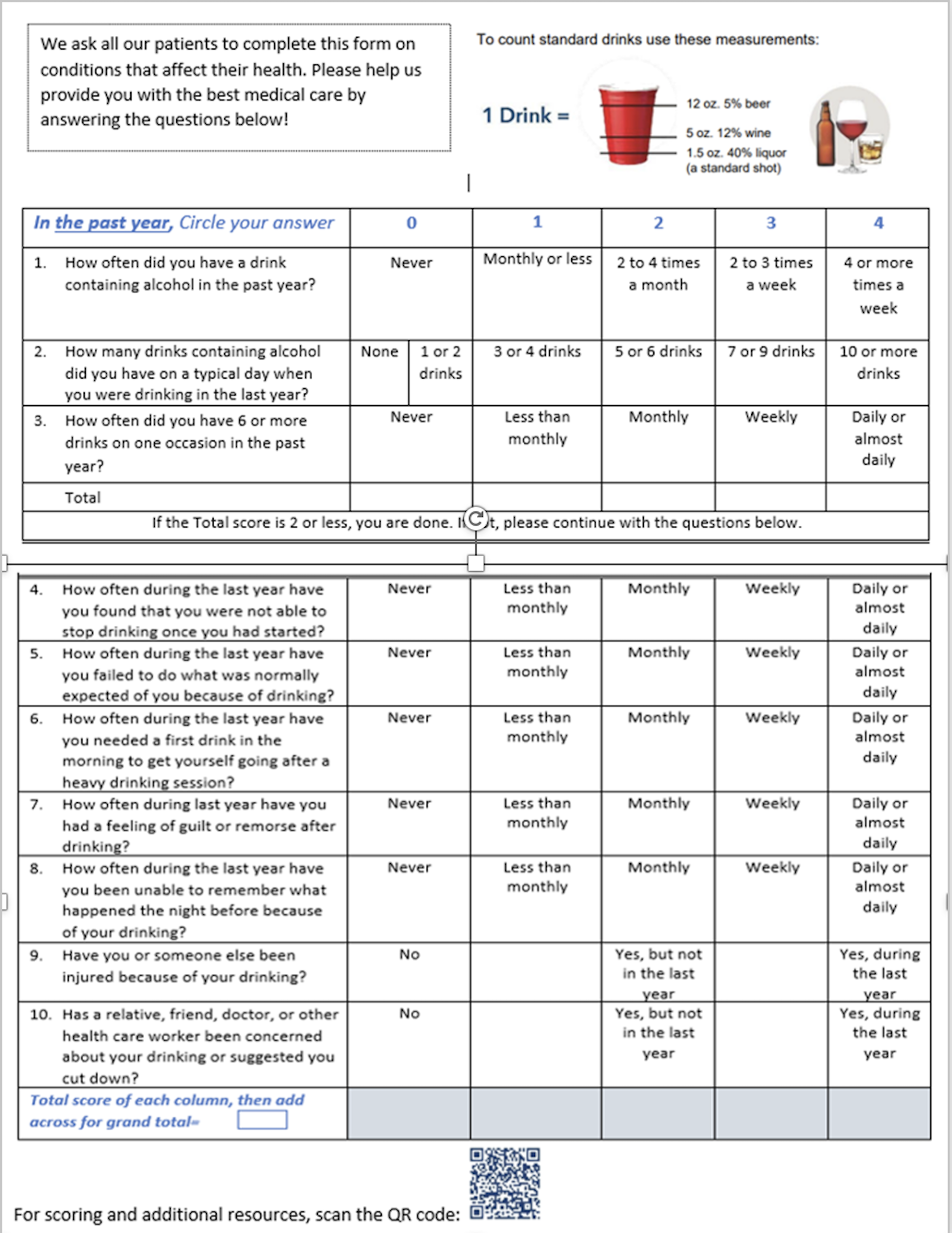

Supplement: Supplementary file 1 — Appendix 1. Adapted AUDIT tool [file 12875_2024_2500_MOESM1_ESM.png]
